# Association between personality traits at return‐to‐work and future work continuation in employees returning from sick leave due to depression: A preliminary study

**DOI:** 10.1002/pcn5.70231

**Published:** 2025-10-26

**Authors:** Eiji Ikeda, Kazumasa Shiozaki, Haruka Ikeda, Takeshi Asami, Yoshio Hirayasu, Akitoyo Hishimoto

**Affiliations:** ^1^ Department of Psychiatry, Graduate School of Medicine Yokohama City University Yokohama Kanagawa Japan; ^2^ Department of Neuropsychiatry, School of Medicine Kanazawa Medical University Kahoku‐gun Ishikawa Japan; ^3^ Yokohama Comprehensive Care Continuum Yokohama Kanagawa Japan; ^4^ School of Humanities Kwansei Gakuin University Nishinomiya Hyogo Japan; ^5^ Yokohama City University Yokohama Kanagawa Japan; ^6^ Hirayasu Hospital Urasoe Okinawa Japan; ^7^ Department of Psychiatry, Graduate School of Medicine Kobe University Kobe Hyogo Japan

Depressive disorders are among the most prevalent mental health problems worldwide. From 1990 to 2021, the age‐standardized prevalence increased by 11.1%, from 3600 to 4007 cases per 100,000 people. Conversely, age‐adjusted disability‐adjusted life years for all causes decreased by 24.7% (from 50,766 to 36,203 per 100,000), whereas those specifically attributable to depressive disorders increased by 13.5%, from 601 to 681 per 100,000.^1^

Depression poses a growing public health concern and significantly affects workforce participation. Meta‐analyses have demonstrated that depressive symptoms increase the chances of taking sick leave by approximately 1.5 times.^2^ Moreover, approximately 40% of workers returning to work following depression‐related sick leave take additional leave within 3 years.^3^ Identifying sustained work predictors at the point of return could facilitate targeted interventions for high‐risk individuals, thereby improving long‐term employment outcomes.

The five‐factor model (FFM) of personality—comprising neuroticism, extraversion, openness, agreeableness, and conscientiousness—has been investigated in relation to return‐to‐work outcomes among employees with depression.^4^, ^5^ Nonetheless, to our knowledge, no study has directly examined how these personality traits, assessed at return to work, are related to long‐term work continuation. We focused on personality traits because they represent relatively stable individual differences that may influence coping, interpersonal functioning, and help‐seeking during the return‐to‐work process. Nonetheless, certain traits, such as extraversion, may be temporarily affected by current clinical states.^6^

This preliminary study explored the association between personality traits assessed at return to work and subsequent continuation of work in a small cohort of public employees. The participants were 15 civil servants (3 men and 12 women; median age, 42 years; interquartile range, 33–46 years) employed by a local government who had returned to work after taking their first leave of absence due to depression. All participants were deemed clinically remitted and fit for return by both their treating psychiatrist and an independent occupational psychiatrist specializing in return‐to‐work evaluations. Individuals with comorbid substance use disorders or signs of manic or hypomanic episodes at assessment were excluded.

Upon return to work, participants completed personality assessments using the taxonomy of the FFM, as measured by the Japanese version of the NEO Five‐Factor Inventory.^7^ Work status was monitored for 3 years. Participants were divided into two groups: the work continuation group (*n* = 9), who remained employed without additional leave or resignation, and the work discontinuation group (*n* = 6), who either took additional sick leave or resigned. Personality trait scores were converted into sex‐adjusted *t*‐scores. Group comparisons were conducted using the Mann–Whitney *U* test with a significance level of 5%. Statistical analyses were performed using spss version 28.

There were no significant differences between groups in terms of age or sex. In the discontinuation group, two‐thirds of individuals experienced work interruption within the first year after returning, with a median duration of work continuation of 6.5 months. Notably, the discontinuation group exhibited significantly higher openness scores than did the continuation group. No significant differences were observed in the other four traits (neuroticism, extraversion, agreeableness, or conscientiousness; Table 1).

**Table 1:** Comparison of demographic characteristics and personality trait scores between work discontinuation and continuation groups.

	Non‐continuers	Continuers	*p*‐value	Effect size
Age	35.5 [27–42.25]	46 [34.5–52]	0.088	0.44
Sex (men, women)	0, 6	3, 6	0.229	0.41
N (T)	57.97 [45.47–66.56]	55.68 [45.38–57.19]	0.456	0.20
E (T)	43.83 [40.5–48.83]	50.5 [42.17–57.25]	0.607	0.15
O (T)	60.70 [58.95–69.04]	51.05 [47.48–59.82]	0.036	0.53
A (T)	57.23 [38.62–64.68]	52.98 [49.26–58.30]	0.689	0.12
C (T)	52.73 [45.45–68.64]	48.18 [44.55–59.09]	0.456	0.21

*Note*: Values are presented as median [interquartile range] unless otherwise indicated. Statistical analyses Fisher's exact test was used for sex, and all other variables were compared using the Mann–Whitney *U* test. Effect size for the Mann–Whitney *U* tests expressed as *r* = *Z*/√*N* (0.10 small, 0.30 medium, and 0.50 large). Effect size for Fisher's exact test expressed as Cramér's *V* = √(*χ*²/[*n* × min(*k* − 1, *r* − 1)]) (0.10 small, 0.30 medium, and 0.50 large).

Abbreviations: A, agreeableness; C, conscientiousness; E, extraversion; N, neuroticism; O, openness; T, *t*‐score.

These findings suggest that greater openness at return to work may be associated with future work discontinuation among individuals recovering from depression. Notably, previous studies have suggested that higher extraversion facilitates continuation following depression.^4^ Nevertheless, our study did not find any significant association with extraversion. This discrepancy may be attributed to differences in assessment timing. While prior research measured personality traits during the sick leave period, our research assessed traits during clinical remission. Because extraversion reduces during severe depression periods,^6^ the participants in our study may have had generally elevated extraversion scores due to symptomatic improvement, which may have obscured any group differences.

Bipolar disorder is frequently misdiagnosed as unipolar depression, with an average diagnostic delay of 8.74 years.^8^ Individuals with bipolar disorder exhibit distinct personality profiles compared to those with unipolar depression, with higher openness scores being the key difference.^8^, ^9^ Our findings, particularly the elevated openness scores in the discontinuation group, may reflect the inclusion of individuals with undiagnosed bipolar disorder. Furthermore, employees with bipolar disorder exhibit significantly higher absenteeism rates compared to controls,^10^ supporting the possibility that unrecognized bipolarity may contribute to poor work continuation outcomes. Nevertheless, this interpretation remains preliminary. Future research should incorporate structured assessments for bipolar disorder to clarify whether unrecognized bipolarity accounts for the poor work continuation outcomes.

This preliminary study has several limitations, including a small sample size and restriction to a single occupational group. Although the proportion of female participants was high, recurrence after a depression‐related sick did not show significant gender differences in prior Japanese research.^3^ Moreover, recurrent sick leave likely also involves clinical and workplace factors, and we did not adjust for multiple comparisons; thus, findings should be interpreted with caution.

Despite these limitations, our study suggests that FFM‐based personality assessment may help identify employees in need of tailored support, with higher openness as a potential marker of risk.

## AUTHOR CONTRIBUTIONS

**Eiji Ikeda**: Conceptualization; data curation; formal analysis; funding acquisition; investigation; methodology; project administration; resources; writing—original draft; writing—review and editing. **Kazumasa Shiozaki**: Investigation; methodology; resources; supervision; writing—review and editing. **Haruka Ikeda**: Investigation; data curation; writing—review and editing. **Takeshi Asami**: Resources; writing—review and editing. **Yoshio Hirayasu**: Resources; supervision; writing—review and editing. **Akitoyo Hishimoto**: Resources; supervision; writing—review and editing. All the authors reviewed the manuscript.

## CONFLICT OF INTEREST STATEMENT

Takeshi Asami is an Editorial Board member of *Psychiatry and Clinical Neurosciences Reports* and a co‐author of this article. To minimize bias, they were excluded from all editorial decision‐making related to the acceptance of this article for publication.

## ETHICS APPROVAL STATEMENT

This study was approved by the Medical Research Ethics Committee of Yokohama City University (approval code: B080911059). All the procedures were performed in accordance with the Declaration of Helsinki.

## PATIENT CONSENT STATEMENT

Before enrollment in the study, each participant was provided with a written explanation of the research protocol, which included an assurance that they would not be disadvantaged if they refused to participate and could withdraw from the study at any time. All the participants provided written informed consent before participating in the study.

## CLINICAL TRIAL REGISTRATION

Not applicable.

## Data Availability

The data that support the findings of this study are available from the corresponding author upon reasonable request.
